# Association Between Vaginal Microbiota and Cervical Dysplasia Due to Persistent Human Papillomavirus Infection: A Systematic Review of Evidence from Shotgun Metagenomic Sequencing Studies

**DOI:** 10.3390/ijms26094258

**Published:** 2025-04-30

**Authors:** Guoda Žukienė, Ramunė Narutytė, Vilius Rudaitis

**Affiliations:** 1 Clinic of Obstetrics and Gynaecology, Faculty of Medicine, Institute of Clinical Medicine, Vilnius University, LT-03101 Vilnius, Lithuania; 2 Faculty of Medicine, Vilnius University, LT-03101 Vilnius, Lithuania

**Keywords:** human papilloma virus, vaginal microbiome, cervical dysplasia, metagenomic shotgun sequencing

## Abstract

The role of vaginal dysbiosis in the progression of human papilloma virus (HPV) associated cervical lesions has gained attention in recent years. While many studies use 16S rRNA gene sequencing for microbiota analysis, shotgun metagenomic sequencing offers higher taxonomic resolution and insights into microbial gene functions and pathways. This systematic review evaluates the relationship between compositional and functional changes in the vaginal microbiome during HPV infection and cervical lesion progression. A literature search was performed according to PRISMA guidelines in PubMed, Web of Science, Scopus, and ScienceDirect databases. Seven studies utilizing metagenomic sequencing in patients with HPV infection or HPV-associated cervical lesions were included. Progression from HPV infection to cervical lesions and cancer was associated with a reduction in *Lactobacillus* species (particularly *Lactobacillus crispatus*) and an enrichment of anaerobic and pathogenic species, especially *Gardnerella vaginalis*. Heterogeneous enriched metabolic pathways were also identified, indicating functional shifts during lesion progression. As most studies were conducted in Asia, further research in diverse regions is needed to improve the generalizability of findings. Future studies employing metagenomic sequencing may help identify biomarkers for early pre-cancerous lesions and clarify the role of vaginal microbiota in persistent HPV infection and cervical dysplasia.

## 1. Introduction

The role of vaginal dysbiosis in human papillomavirus (HPV) infection-associated cervical lesions has been an important topic of research in recent years. Substantial evidence has been emerging to support the connection between vaginal dysbiosis and cervical intraepithelial neoplasia (CIN) or cervical cancer (CC) [1,2,3,4,5].

The normal vaginal microbiome is dominated by *Lactobacillus* genus [6,7,8]. Several confounding factors influence vaginal microbiota composition, including recent antibiotic or probiotic use, sexual activity, menstrual cycle phase, and hygiene practices [9,10]. Microbiota profiles are also associated with age, ethnicity, parity, menopausal status, multiple sexual partners, HPV vaccination, hormonal contraceptive use, estrogen levels, and BMI [10,11].

The predominant *Lactobacilli* offer several host benefits, primarily by creating an acidic environment that inhibits inflammation and creating a biofilm that inhibits pathogen growth [12]. Additionally, *Lactobacilli* have been found to produce anti-inflammatory compounds [13] and control host/immune response by interacting selectively with a subset of anti-inflammatory receptors through their surface layer proteins [14]. Decreased *Lactobacillus* spp. enrichment and increased enrichment of anaerobic bacterial vaginosis (BV)-associated species has been linked to HPV infection and cervical pre-cancerous lesion progression, prompting research into microbiota as potential biomarkers for early HPV-associated lesions [3,15]. Other research has linked a *Lactobacillus*-depleted, anaerobe-rich cervicovaginal microbiome to a pro-inflammatory environment and an elevated risk of preterm birth or acquiring sexually transmitted diseases [14,16,17].

Most studies investigating the vaginal microbiome rely on 16S rRNA gene amplification [2,3,4,5,18]. Compared to 16s rRNA sequencing, shotgun metagenomic sequencing is costlier and more complex, although it provides higher taxonomic resolution—down to the strain level [19]—and offers insights into gene functions and metabolic pathways relevant to the tumor microenvironment [7,8,20]. It has been shown to outperform 16S rRNA sequencing at higher taxonomic levels, identifying significantly more species (1174 vs. 304), thus confirming its greater sensitivity [19]. The application of metagenomic shotgun sequencing enables a more comprehensive analysis of microbiota functional mechanisms involved in cervical lesion development during HPV infection. This approach captures a broader range of species associated with cervical dysplasia, facilitating the identification of novel biomarkers that could improve diagnostic accuracy and provide critical insights into the potential protective role of the vaginal microbiota in cervical lesion progression. Therefore, the aim of this systematic review is to assess the relationship between compositional and functional changes in the vaginal microbiome and the progression of cervical dysplasia due to persistent HPV infection based on studies employing metagenomic shotgun sequencing.

## 2. Methods

### 2.1. Search Strategy and Study Selection

This systematic review was conducted using the PICO (Patient, Intervention, Comparison, Outcome) framework [21] and in accordance with the Preferred Reporting Items for Systematic Reviews and Meta-Analyses (PRISMA) guidelines [22]. The population included women with histologically confirmed cervical dysplasia or confirmed HPV infection. Vaginal microbiome composition was assessed via shotgun metagenomic sequencing, with comparisons to women without dysplasia. The primary outcome was to assess the association between the composition of vaginal microbiota and HPV persistence or the progression of cervical dysplasia. The secondary outcome was to assess the functional changes of vaginal microbiota during HPV infection and cervical dysplasia.

Studies were included if they employed shotgun metagenomic sequencing to analyze vaginal microbiota in women with HPV-associated dysplasia, were peer-reviewed, published between 2007 and 2024, and written in English. Studies utilizing alternative sequencing methods, those not focused on cervical dysplasia or HPV infection, as well as reviews and case reports, were excluded.

A comprehensive literature search was conducted in PubMed, Web of Science, Scopus, and ScienceDirect using relevant search terms for vaginal microbiota, cervical dysplasia, HPV, and metagenomics (search strategy provided in Supplementary Materials, Table S3). The search was last updated on 1 December 2024.

**Figure 1:**
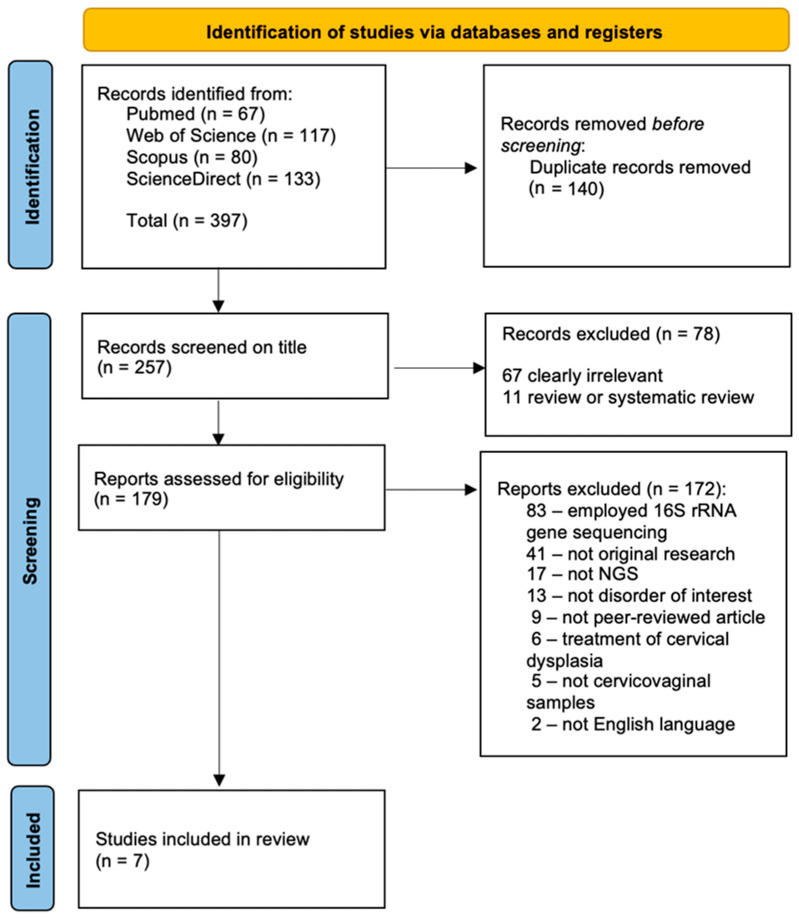
PRISMA flowchart.

Titles and abstracts were initially screened by two independent reviewers (G.Ž. and R.N.), followed by a full-text assessment to determine eligibility. Seven studies [6,7,8,19,20,23,24] met the eligibility criteria. The PRISMA flowchart is provided in the Results and Discussion section (Figure 1). This review was registered in the Research Registry database under the registration number reviewregistry1985.

### 2.2. Data Extraction

Data extraction was carried out by two independent reviewers (G.Ž. and R.N.) and included study characteristics (such as author, year, country, and study period), population characteristics (including demographics and recruitment methods), as well as data on vaginal microbiome composition (phyla, genus, and species-level diversity, measured by Shannon or Simpson indices and relative abundance) in relation to HPV infection (any HPV or high-risk HPV), as well as cervical dysplasia or cervical cancer (based on histological findings). Additionally, data from four studies [7,19,20,23] on differentially enriched genes annotated to biochemical functions via the Kyoto Encyclopedia of Genes and Genomes (KEGG) were collected. The data on diversity and relative abundances of taxonomic assignments, both within and across disease states, were qualitatively synthesized and categorized as either significantly differentially increased or decreased in comparison to healthy controls.

### 2.3. Quality Assessment

**Figure 2:**
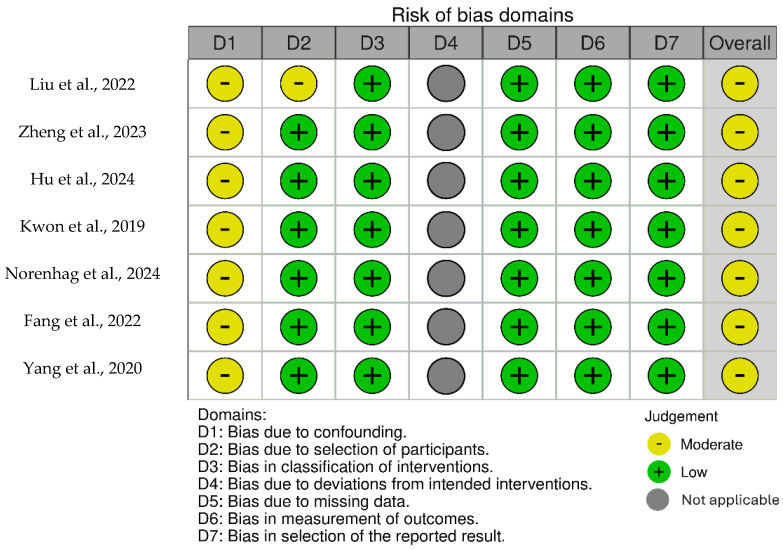
Risk of bias assessment [6,7,8,19,20,23,24].

The risk of bias for all included studies was assessed using a modified version of the ROBINS-I tool, tailored to accommodate the design and characteristics of cross-sectional studies (Figure 2), by two independent reviewers (R.N. and G.Ž.), with discrepancies resolved through discussion. Domains assessed included confounding, participant selection, exposure classification, missing data, outcome measurement, and result selection. Judgments were categorized as “low risk”, “moderate risk”, “serious risk”, or “critical risk”. All studies were assessed as having a moderate risk of bias, mainly due to insufficient adjustment for confounding factors. None of the selected studies had a registered protocol, making it difficult to determine if selective reporting occurred. However, since the studies aimed to comprehensively assess the vaginal microbiome and showed no indication of selective reporting, the risk of bias in this regard was considered low. The risk of bias assessment is summarized in Figure 2, with a detailed evaluation of each domain provided in Supplementary Materials, Table S2.

## 3. Results and Discussion

### 3.1. Study Selection and Characteristics

The study selection was conducted in accordance with PRISMA guidelines (Figure 1).

The characteristics of selected studies are presented in Table 1. Overall, seven studies were selected: two comparing patients with HPV to healthy controls [6,19], one study comparing healthy, HPV-positive, CIN, and CC groups [7], two studies comparing healthy, CIN, and CC groups, and subdividing patients to HPV-positive and -negative groups [23,24], and one study comparing healthy, CIN, and CC groups [20]. Five studies were based in China [6,7,8,19,24], one in South Korea [20], and one in Sweden [23]. All studies utilized shotgun metagenomic sequencing, and one additionally utilized the 16S rRNA sequencing method for comparison purposes [19]. Six studies used patients without cervical lesions or HPV infection (normal cervical (NC) group) as the control [6,7,19,20,23,24], while one study used HPV-positive patients as the control group [8].

### 3.2. Differences in Microbiota Diversity

One out of four studies comparing phyla- and genus-level diversity calculated by Shannon or Simpson indices in high-risk HPV (HR-HPV) infected patients and controls reported increased diversity in women with HR-HPV [19], while two others found no significant difference in phyla- and genus-level diversity between healthy individuals and those with pre-cancerous lesions [6,20]. At the species level, two studies showed higher microbiota diversity in HPV-positive women [8,24], while two others observed no significant differences compared to HPV-negative individuals [6,19]. Of four studies assessing microbial diversity in cervical dysplasia, one found no significant difference, while three reported higher diversity in dysplasia patients [8,23,24], with two showing significant increases as lesions progressed [8,24].

### 3.3. Differences at the Phylum Level

Firmicutes and Actinobacteriota were reported as the predominant phyla in cervicovaginal microbiota [6,19]. At the phylum level, Firmicutes were reported as more abundant in healthy individuals, whereas Actinobacteriota, Fusobacteria, and viruses showed significant enrichment in patients with HR-HPV [6,19]. One study found that the abundance of Firmicutes, Proteobacteria, and Planctomycetes decreases in cervical cancer (CC) patients compared to those with CIN2/3, while Spirochaetes showed an increased prevalence as cervical lesions progressed [20].

### 3.4. Differences at the Genus Level

The most abundant genera in cervicovaginal microbiota include *Lactobacillus, Leptospira, Gardnerella, Ehrlichia, Clostridium*, and *Streptococcus* [7,20]. In healthy individuals, *Lactobacilli* dominate the microbiota [6,7,8], comprising 84.90%, with lower abundances of *Gardnerella* (1.76%), *Atopobium* (0.21%), and *Bifidobacterium* (0.34%) [19]. In contrast, patients with HPV show a reduced *Lactobacillus* abundance (54.98–65.96%) and increased abundance of *Gardnerella* (7.81–11.59%), *Atopobium* (3.43%), *Bifidobacterium* (8.76%), *Peptostreptococcus*, and *Prevotella* [6,8,19]. Three studies reported that the abundance of *Lactobacillus* also decreases with the progression of cervical lesions [7,8,20], while genera such as *Gardnerella, Prevotella, Staphylococcus, Candidatus Endolissoclinum, Alkaliphilus, Pseudothermotoga*, and *Wolbachia* showed increased abundance in individuals with cervical lesions or cervical cancer [8,20]. *Lactobacillus* genus has been found to be a highly significant biomarker for distinguishing tumor (CC or CIN) and non-tumor (HPV or healthy) patients, including the most influential biomarkers, *L. iners*, *L. crispatus*, and *G. vaginalis* [7].

### 3.5. Differences at Species Level

As for species, *L. crispatus*, *L. iners*, *L. gasseri*, and *L. jensenii* are the most abundant in healthy individuals [8,19,23,24]. Differences in the microbiome in HPV-infected, CIN, and CC patients are presented in Table 2.

One study evaluating vaginal community state types (CST) reported that CST I, II, and III (dominated by *L. crispatus*, *L. gasseri*, and *L. iners*, respectively) are the most common in healthy controls (42.4%, 31%, and 24.3%, respectively) [23]. Five studies indicated a decrease in the abundance of all *Lactobacilli*, particularly *L. crispatus*, and an increase in *G. vaginalis* abundance in the presence of cervical lesions or HPV infection [7,8,19,23,24]. Studies comparing HR-HPV-positive patients and controls confirm this tendency, as three studies found *L. crispatus* to be dominant in HPV-negative individuals [19,23,24], while five studies reported that *G. vaginalis* is significantly enriched in HR-HPV-positive patients [6,7,8,19,23]. The microbiota of HPV-positive patients has been found to exhibit a significantly lower abundance of *L. jensenii*, *L. helveticus* [19], *L. gasseri* [23], and *L. iners* [8]. In contrast, one study found no significant difference in *L. crispatus* between healthy women and those with HPV16 [6]. Another study reported that no microbiota differences were observed between HPV-negative and low-risk HPV-infected women [23], suggesting microbiota changes occur primarily in HR-HPV cases [23]. In patients with CIN 2, 3, or CC, a decreased abundance of *L. crispatus* [7,8,24] and *L. iners* [8,24] is observed. Enrichment of *L. crispatus* and *L. iners* has been found to decrease significantly with the progression of dysplasia [8]. Interestingly, three studies observed that as cervical lesions progress and the overall abundance of *Lactobacillus* spp. declines, *L. crispatus* [7,8,23], *L. gasseri*, and *L. jensenii* [7] decrease more relative to *L. iners*, leading to the increased predominance of *L. iners* [7,8,23]. A study comparing CST between healthy controls and patients with dysplasia showed that CST I, dominated by *L. crispatus*, was less prevalent in dysplasia patients (24.3%) than in controls (42.4%), while CST III, dominated by *L. iners*, was more frequent in the dysplasia group (26% vs. 20.3%) [23]. These findings suggest that both the abundance and composition of *Lactobacillus* spp. play a role in dysplasia development.

The progression of cervical lesions is marked by a reduction in CST types I through III and an increase in CST IV, which is rich in anaerobic bacteria [8,23]. CST IV is the most prevalent type in women with dysplasia, accounting for 44.6% [23]. Although *G. vaginalis* is the most common anaerobe significantly increased in patients with HR-HPV [7,8,19,23,24], notable increases in other opportunistic pathogens, such as *Gardnerella* spp. 304 and 2612, *Peptostreptococcus anaerobius*, *Mobiluncus curtisii*, *Prevotella* spp., and *Fusobacterium nucleatum* [6], *Peptoniphilus lacrimalis*, *Fannyhessae vaginae*, and *Mageeibacillus indolicus* [23], as well as a decrease in *Enterococcus* sp. 1140_ESPC [6] are observed in HR-HPV-positive women. One study reported that non-bacterial biomarkers, including *Methanobrevibacter oralis* (archaea), *Candida albicans* (eukaryote), and *Alpha papillomavirus 9* (virus), were enriched in HPV16-positive women, suggesting the role of non-bacterial taxa in HR-HPV infection [6]. Further increases in *G. vaginalis* [7,8,23,24] and other anaerobes such as *Aerococcus christensenii*, *Peptoniphilus lacrimalis*, and *Fannyhessae vaginae* [23] are reported in patients with cervical lesions. *Fannyhessae vaginae* (*Atopobium vaginae*), an anaerobe linked to BV, shows conflicting evidence; one study reported decreased enrichment in CIN and CC patients [7], while another found significant enrichment in dysplasia patients [23]. Interestingly, two studies observed that, although *G. vaginalis* is enriched in patients with HPV positivity and CIN, its abundance declines in those with CC [7,8]. Additionally, a variety of pathogenic bacteria, such as *Staphylococcus aureus*, *Phocaeicolavulgatus*, *Salmonella enterica*, *B. fragilis*, and *Prevotella bivia*, show significant increases in CC patients [8,24].

### 3.6. Functional Differences of Cervicovaginal Microbiome

Four studies investigated the functional pathways of the cervicovaginal microbiome, revealing distinct functional differences in patients with HPV infection, CIN, and CC [7,19,20,23]. These pathways were categorized using the Kyoto Encyclopedia of Genes and Genomes (KEGG) classification. Enriched pathway categories in different stages of cervical lesions are summarized in Table 3, and specific enriched pathways are provided in Supplementary Material Table S1.

Pathways related to lysine [20,23], other amino acid (L-threonine, L-methionine) [23], and carbohydrate [19,20,23] metabolism are enriched in healthy individuals, mainly driven by *L. crispatus*, *L. iners*, and *L. jensenii* [23]. Enriched pathways in the metabolism of cofactors, vitamins, and xenobiotics were also observed in patients without cervical lesions, primarily driven by *L. crispatus*, *L. iners*, *L. jensenii*, *F. vaginae*, and *G. vaginalis* [23]. Pathways related to carbohydrate metabolism, genetic processing, and membrane transport were notably abundant in HPV-infected patients [7,19]. Enrichment of biosynthesis pathways for L-alanine, L-valine, L-isoleucine, and aromatic amino acids was observed in patients with dysplasia, primarily driven by *G. vaginalis*, *Bifidobacterium longum*, and other unclassified bacteria [23]. Nucleotide metabolism pathways were significantly enriched in dysplasia patients, with *G. vaginalis* and *F. vaginae* as key contributors [23]. Two studies reported enrichment in peptidoglycan biosynthesis in dysplasia or CC patients, with *G. vaginalis*, *P. bivia*, *P. timonensis*, *Streptococcus agalactiae*, and *S. anginosus* as main contributors [20,23]. This pathway was not enriched in HPV-only patients [19].

### 3.7. Discussion

This systematic review highlights the role of microbiota changes in persistent HPV infection and cervical pre-cancerous lesion progression. Several studies have reported increased diversity of cervicovaginal microbiota in patients with HPV infection and HPV-associated cervical lesions [8,19,23,24], which is in line with previously published literature [1,2,18]. Our findings of reduced *Lactobacillus* spp. and increased anaerobic bacteria, particularly *G. vaginalis* and other BV-associated species, are consistent with existing literature, supporting a link between the vaginal microbiome and HPV-associated cervical lesions [1,2,3,4,5]. One of the selected studies reported strong correlations between pathogenic genera (e.g., *Sneathia*, *Salmonella*, *Leptotrichia*) and HPV E6/E7 oncogene overexpression, with *S. amnii*, *S. enterica*, and *E. faecalis* showing the strongest associations. Additional biomarkers identified included *Chlamydia trachomatis*, *Veillonella montpellierensis*, and *Bifidobacterium* spp. [8]. Another study found that cervicovaginal dysbiosis increases unmethylated cytosine–phosphate–guanine (CpG) motifs, promoting toll-like receptor 9 (TLR9) expression and advancing cervical lesion progression [24]. TLR9, a pattern recognition receptor, detects microbial DNA and triggers immune responses. Higher TLR9 expression was observed in HPV-positive women and correlated with microbiota diversity, particularly *G. vaginalis* dominance, as well as *S. amnii* and *P. vulgatus*. In contrast, lower expression was associated with *L. crispatus* dominance [24]. Clinical BV symptoms were associated with high-grade squamous intraepithelial lesions (HSIL) and HR-HPV infection, with specific pathogens (*P. lacrimalis*, *G. vaginalis*, *F. vaginae*) linked to abnormal discharge in HR-HPV-infected patients [23]. While *G. vaginalis* is widely associated with HR-HPV infection and cervical lesions [7,8,19,23,24], other significantly enriched species vary across studies, making it challenging to identify consistent biomarkers. Expanding the use of highly sensitive metagenomic shotgun sequencing in future research may offer a more comprehensive understanding of additional species potentially involved in persistent HPV infection and cervical lesion development. *Lactobacilli*, particularly *L. crispatus*, have been identified as protective against HPV infection [3,15]. Consistent with our findings, other studies report a more significant decrease in *L. crispatus* in HPV-infected patients, while *L. iners* is more prevalent in those with cervical lesions [1,3,15]. These findings suggest that not all *Lactobacillus* species may be of equal importance in preventing or clearing HPV infection [1,3,15,18].

Differences in microbiome functional pathways among patients with HPV infection and varying cervical lesion severity were reported by several authors, indicating changes in microbial function during cervical lesion progression [7,16,17,19]. Pathways associated with amino acid [17,19], carbohydrate [16,17,19], cofactor, vitamin, and xenobiotic metabolism are enriched in healthy individuals, primarily driven by *Lactobacillus* species. In contrast, patients with dysplasia or CC exhibit more enriched pathways related to nucleotide metabolism and peptidoglycan synthesis, with *G. vaginalis*, *F. vaginae*, *B. longum*, *P. bivia*, *P. timonensis*, *S. agalactae*, and *S. anginosus* as key contributors. One study investigating genetic mutations, particularly single nucleotide variants (SNVs), found that *G. vaginalis* mutations showed an increased prevalence in CC and CIN groups, while *Lactobacillus* spp. and *S. agalactiae* mutations were more commonly found in HPV-positive and control groups. Additionally, diagnostic models based on SNV counts across species outperformed species abundance models, suggesting SNVs as promising biomarkers for early cervical lesions [7]. However, further studies are required to confirm their diagnostic utility in HPV-associated cervical lesions.

A comprehensive understanding of the microbiota’s role in HPV-related lesion progression may be clinically relevant for several reasons. Women with BV-associated microbiota compositions may require more frequent monitoring of cervical lesions. The link between BV-associated species and HR-HPV-induced dysplasia suggests that timely diagnosis and treatment of BV may mitigate the risk of dysplasia development. Moreover, lifestyle modifications that support a favorable microbiota may protect against HPV infection or enhance HPV clearance. The possible protective role of a healthy vaginal microbiome in HPV infection has driven research into the use of probiotics and prebiotics to improve vaginal health and promote HPV clearance. One pilot trial inspecting intravaginal transplantation of a vaginal isolated natural probiotic strain, *Lactobacillus crispatus*, showed significantly reduced viral load of HR-HPV, ameliorated HR-HPV clearance rate, and improved vaginal inflammation state [25]. However, robust evidence from high-quality, randomized, placebo-controlled trials remains lacking [26].

The main limitation of this systematic review is the absence of a meta-analysis, with the included studies reporting heterogeneous species enrichment in HPV-infected patients and those with cervical dysplasia or cancer. However, a clear trend of increased anaerobe and decreased *Lactobacillus* enrichment is observed. Meta-analyses of research employing highly sensitive metagenomic sequencing are needed to identify species associated with persistent HPV infection. Further research should clarify the role of microbiome functional pathway changes during HPV infection and cervical lesion progression while also including demographic and lifestyle data, applying multivariable statistical models, and identifying key confounding factors affecting cervicovaginal microbiome composition. Bias may occur due to the regional and ethnic heterogeneity of vaginal microbiome samples, as most studies, with the exception of Norenhag et al., 2024 [23], focused on Chinese [6,7,8,19,24] or South Korean [20] populations. Given the limited use of metagenomic sequencing and potential ethnic differences in microbiota, we reviewed some studies analyzing microbiota across regions using 16S rRNA sequencing. *L. iners* tends to dominate over *L. crispatus* in Latina [1], African [9], and Costa Rican [27] women, whereas *L. crispatus*-dominated CSTs are more prevalent in European [10] and US [11] populations. A study from Africa reported that most women had non-*Lactobacillus*-dominated cervical microbiota [11], which may contribute to the higher prevalence and slower clearance of HR-HPV observed in African women compared to their European counterparts [28]. Despite regional differences in microbiome composition, multiple studies [1,27] and meta-analyses [3,4] from diverse geographical regions consistently report that women with HPV, particularly HR-HPV, are more likely to exhibit low-*Lactobacillus* CSTs, vaginal dysbiosis, and increased microbial diversity [1,4,23,27].

Additionally, the scarcity of studies investigating genetic and metabolic changes in the cervicovaginal microbiota, along with heterogeneous pathway enrichment, limits conclusions on specific pathways. This underscores the need for further research on functional changes in the vaginal microbiome to clarify their role in HPV-associated cervical lesions.

## 4. Conclusions

This systematic review provides insights into microbiota diversity and its role in HPV infection and cervical lesion progression. High-risk human papillomavirus (HPV)-infected patients show increased cervicovaginal microbiota diversity, which rises with lesion progression. HPV-positive patients and those with cervical lesions exhibit reduced *Lactobacillus* spp. and increased anaerobes, including *Gardnerella* and *Prevotella*. *Lactobacillus crispatus* is strongly linked to healthy states, while *Lactobacillus iners* remains prevalent in progressing lesions. *Gardnerella vaginalis* is commonly enriched in HPV infections and cervical intraepithelial neoplasia (CIN), but its enrichment has been reported to decrease as CIN progresses to cancer, with other anaerobic and pathogenic species becoming more abundant. Metagenomic shotgun sequencing has identified genetic and metabolic microbiome alterations linked to dysplasia progression. However, further research is needed to fully clarify their role in HPV-associated cervical lesions.

## Figures and Tables

**Table 1:** Characteristics of selected studies.

1st Author, Year	Country	Study Design	Population	HPV Genotypes	*N*	Groups	Primary Comparator	Type of Sequencing
Liu et al., 2022 [8]	China	Observational, cross-sectional	Patients diagnosed with HPV, CIN, or CC (histologically verified) for the first time.	High risk—16, 18, 26, 31, 33, 35, 39, 45, 51, 52, 53, 56, 58, 59, 66, 68, 82	115	HR-HPV without CIN (*n* = 34) CIN with HR-HPV (*n* = 40) CC (*n* = 41)	HR-HPV group	Metagenomic shotgun sequencing
Zheng et al., 2023 [24]	China	Observational, cross-sectional	Patients with histologically verified CIN1, CIN2/3, squamous CC, and healthy controls.	16	341	CIN1 (*n* = 90) CIN2/3 (*n* = 78) CC (*n* = 49) NC (*n* = 124) Subdivision to HPV16-positive (*n* = 128), other HPV-positive (*n* = 34) and HPV-negative (*n* = 179)	NC group	Metagenomic shotgun sequencing
Hu et al., 2024 [7]	China	Retrospective observational cohort	HPV-positive patients and patients with histologically verified CIN, CC, and healthy controls.	Not specified	151	CC (*n* = 42) CIN (*n* = 43) HPV+ (*n* = 34) NC (*n* = 32)	NC group	Metagenomic shotgun sequencing
Kwon et al., 2019 [20]	South Korea	Observational, cross-sectional	Patients with histologically verified CIN or CC and healthy controls.	-	47	CIN 2/3 (*n* = 17) CC (*n* = 12) NC (*n* = 18)	NC group	Metagenomic shotgun sequencing
Norenhag et al., 2024 [23]	Sweden	Observational, cross-sectional	Patients with histologically verified dysplasia and cancer (LSIL, HSIL, CC) and healthy controls.	Low risk—6, 11, 32, 34, 37, 40, 42, 43, 44, 54, 61, 62, 70, 71, 72, 74, 80, 81, 83, 84, 87, 89, 90, 91, 98, 101, 103, 106, 107, 108, 114, 115, 118, 119, 121, 124, 129, 149, 155, 163, 168 High risk—16, 18, 31, 33, 35, 39, 45, 51, 52, 56, 58, 59, 68, 73, 82	354	Dysplasia and cancer (*n* = 177; (LSIL *n* = 81, HSIL *n* = 94, cancer *n* = 2)) NC (*n* = 177) Subdivision to HPV-negative, (*n* = 35), LR-HPV (*n* = 26), HR-HPV (*n* = 126)	NC group	Metagenomic shotgun sequencing
Fang et al., 2022 [19]	China	Observational, cross-sectional	Reproductive-age women with HR-HPV and healthy controls.	Not specified	40	HR-HPV (*n* = 20) NC (*n* = 20)	NC group	16S rRNA gene and shotgun metagenomic sequencing
Yang et al., 2020 [6]	China	Observational, cross-sectional	Reproductive-age women with HPV16 and healthy controls.	16	57	HPV16 positive (*n* = 27) NC (*n* = 25)	NC group	Metagenomic shotgun sequencing

Abbreviations: NC—normal cervical group; HPV—human papilloma virus; CIN—cervical epithelial neoplasia; CC—cervical cancer; HR—high risk; LR—low risk; HSIL—high-grade squamous intraepithelial lesion; LSIL—low-grade squamous intraepithelial lesion.

**Table 2:** Species Abundance Variations in HPV, CIN, or CC Compared to Healthy Individuals.

Species	HPV					Dysplasia				CC			Aerobic/Anaerobic Status	BV-Related Organisms
	Author, Year					Author, Year				Author, Year				
	Zheng et al., 2023 [24]	Hu et al., 2024 [7]	Norenhag et al., 2024 [23]	Fang et al., 2022 [19]	Yang et al., 2020 [6]	Liu et al., 2022 [8] **	Zheng et al., 2023 [24]	Hu et al., 2024 [7]	Norenhag et al., 2024 [23]	Liu et al., 2022 ** [8]	Zheng et al., 2023 [24]	Hu et al., 2024 [7]		
*Lactobacillus crispatus*	↓			↓		↓	↓	↓	↓	↓	↓	↓	Fan	
*Lactobacillus iners*	↓	↓				↓	↓	↓		↓	↓	↓	Fan	
*Lactobacillus gasseri*			↓	↓		↓		↓		↓		↓	Fan	
*Lactobacillus jensenii*	↓	↓		↓		↓		↓		↓		↓	Fan	
*Lactobacillus helveticus*				↓									Fan	
*Lactobacillus acidophilus*						↓				↓			Fan	
*Lactobacillus johnsonii*				↑									Fan	
*Gardnerella vaginalis*	↑	↑	↑	↑	↑	↑	↑	↑	↑	↑	↓	↓	An	BV
*Gardnerellasp_304*					↑								An	BV
*Gardnerella sp_2612*					↑								An	BV
*Bifidobacterium breve*		↓		↑			↑	↑				↑	An	
*Bifidobacterium longum*						↑				↑			An	
*Bifidobacterium bifidum*				↑									An	
*Prevotella bivia*		↓			↑	↑		↑		↑		↑	An	BV
*Prevotella amnii*		↓			↑	↑		↓		↑		↓	An	BV
*Prevotella corporis*					↑								An	
*Prevotella disiens*					↑								An	BV
*Prevotella timonensis*		↓						↓				↓	An	
*Peptoniphilus lacrimalis*			↑										An	
*Peptoniphilus harei*											↑		An	BV
*Mageeibacillus indolicus*			↑										An	
*Atopobium vaginae/Fannyhessea vaginae*		↓	↑					↓	↑			↓	An	BV
*Mobiluncus curtisii*					↑								An	BV
*Coriobacteriales bacterium DNF00809*					↑								An	
*Peptostreptococcus anaerobius*					↑								An	BV
*Veillonella montpellierensis*					↑	↑				↑			An	
*Megasphaera* sp. *UPII_135E*					↑								An	BV
*Fusobacterium nucleatum*					↑								An	BV
*Methanobrevibacter oralis*					↑								An	
*Finegoldia magna*												↑	An	
*Porphyromonas uneanis*		↓						↑				↑	An	
*Porphyromonas asaccharolytica*							↑				↑		An	
*Snethia amnii*							↑				↑		An	BV
*Phocaeicola vulgatus*											↑		An	
*Bacteroides fragilis*						↑	↑			↑	↑		An	
*Bacteroides thetaiotaomicron*											↑		An	
*Clostridium botulinum*											↑		An	
*Anaerococcus lactolyticus*						↑				↑			An	
*Anaerococcus tetradius*						↑				↑			An	
*Peptoniphilus lacrimalis*									↑				An	
*Burkholderia pseudomallei*				↑									A	
*Ureaplasma parvum*								↑					A	
*Neisseria gonorrhoeae*											↑		A	
*Bacillus velezensis*						↑				↑			A	
*Aerococcus christensenii*									↑				A	
*Klebsiella pneumonia*				↑									Fan	
*Escherichia coli*		↓				↓		↓		↓	↑	↓	Fan	
*Streptococcus agalacitae*		↓						↑				↓	Fan	
*Streptococcus mitis oralis pneumoniae*		↑						↑				↑	Fan	
*Staphylococcus aureus*	↑						↑				↑		Fan	
*Salmonela enterica*							↑				↑		Fan	
*Streptococcus mitis*						↓				↓			Fan	
*Candida albicans*					↑								A, fungus	
*Alpha papillomavirus 9*					↑								Virus	

Abbreviations: HPV—human papilloma virus; CC—cervical cancer; A—aerobe; An—anaerobe; Fan—facultative anaerobe; BV—bacterial vaginosis. ↑—significantly increased enrichment compared to primary comparator; ↓—significantly decreased enrichment compared to primary comparator. ** HPV-infected patients without cervical lesions served as the primary comparator in this study.

**Table 3:** Enriched functional pathways of cervicovaginal microbiota.

Group	Enriched Pathway	Contributing Species
Healthy	Aminoacid metabolism [20,23]; Carbohydrate metabolism [19,20,23]; Nucleotide metabolism [23]; Xenobiotics biodegradation and metabolism [19,20,23]; Metabolism of cofactors and vitamins [23]; Cellular processes [20]; Genetic information processing [19,23]; Signal transduction [7,20]; Membrane transport [19]; Human diseases [19]; Biosynthesis of other secondary metabolites [19].	*L. crispatus* (mainly), *L. jensenii*, *L. iners*, *L. rhamnosus*, *G. vaginalis*, *F. vaginae* [23]
HPV	Carbohydrate metabolism [19]; Genetic information processing [7,19]; Membrane transport [19].	
CIN	Aminoacid metabolism [20,23]; Carbohydrate metabolism [23]; Nucleotide metabolism [23]; Peptidoglycan synthesis [20,23]; Genetic information processing [7]; Human diseases [19].	*G. vaginalis*, *B. longum*, *F. vaginae*, *P. bivia*, *P. timonensis*, *S. agalactiae*, *S. anginosus* [23]
Cervical cancer	Peptidoglycan synthesis [20]; Genetic information processing [7]; Signal transduction [7]; Human diseases [19].	

## Supplementary Materials

The following supporting information can be downloaded at: https://www.mdpi.com/article/10.3390/ijms26094258/s1.

## Abbreviations

The following abbreviations are used in this manuscript:

HPV	Human papilloma virus
CIN	Cervical intraepithelial neoplasia
CC	Cervical cancer
