# Supplementary material for: Association Between Vaginal Microbiota and Cervical Dysplasia Due to Persistent Human Papillomavirus Infection: A Systematic Review of Evidence from Shotgun Metagenomic Sequencing Studies
Source: Int J Mol Sci. 2025 Apr 30;26(9):4258. doi: 10.3390/ijms26094258 (PMC12072622; doi:10.3390/ijms26094258)
Supplement: Supplementary file 1 [file ijms-26-04258-s001.zip › ijms-3549598-supplementary.pdf]

Supplementary materials

Enriched functional pathways of cervicovaginal microbiota in healthy individuals, HPV infection, cervical intraepithelial neoplasia and cervical cancer

Table S1. Enriched functional pathways of cervicovaginal microbiota in healthy individuals, HPV infection, cervical intraepithelial neoplasia and cervical cancer.

Metabolic pathway	Author, year											Main contributing species (Norenhag et al, 2024) [20]	Type of metabolic pathway
	NC				HPV		Dysplasia			CC			
	Kwon et al, 2019 [17]	Fang et al, 2022 [16]	Hu et al, 2024 [7]	Norenhag et al, 2024[20]	Fang et al, 2022 [16]	Hu et al, 2024 [7]	Kwon et al, 2019 [17]	Norenhag et al, 2024[20]	Hu et al, 2024 [7]	Kwon et al, 2019 [17]	Hu et al, 2024 [7]		
Lysine biosynthesis (ko00300)	↑			↑			↑					*L. crispatus*	Amino Acid Metabolism
Biosynthesis of flavin, L-threonine, L-methionine (RIBOSYN2-PWY, THRESYN-PWY, PWY-724)				↑								*L. crispatus*	
L-alanine biosynthesis (PWY0-1061)								↑				*G. vaginalis*	
L-valine (VALSYN-PWY)								↑				*Bifidobacterium longum, unclassified taxa*	
Aromatic amino-acid (COMPLETE-ARO-PWY)								↑				*Bifidobacterium longum, unclassified taxa*	
L-isoleucine biosynthesis (PWY-3001)								↑				*Bifidobacterium longum, unclassified taxa*	
Cyanoamino acid metabolism (ko00460)	↑												
N-acetyl-glucosamine, -mannosamine and -neruaminate degradation (GLCMANNANAUT-PWY)				↑								*L. crispatus*	
Methane metabolism (ko00680)	↑						↑						
Peptidoglycan biosynthesis and maturation (ko00550, PWY-5265, PWY-6385, PWY0-1586)					ND		↑	↑		↑		*G. vaginalis, P. bivia, P. timonensis, S. agalactae, S. anginosus*	Biosynthesis of Other Secondary Metabolites
Biosynthesis of vancomycin group antibiotics (ko01055)		↑											
Galactose metabolism (ko00052)		↑											Carbohydrate Metabolism
Amino sugar and nucleotide sugar metabolism (ko00520)		↑			↑								
Pyruvate metabolism, Pyruvate fermentation to acetate and lactate II (PWY-5100)	↑			↑								*L. crispatus, L. iners*	
Starch metabolism	↑												
Carbohydrate metabolism					↑								
Sucrose metabolism	↑												

Galactose and sucrose degradation (PWY66-422, PWY-6317, PWY-621)				↑								*L. jensenii*	
Starch degradation V (PWY-6737)								↑				*F. vaginae*	
Biofilm formation in Vibrio cholerae (ko05111)	↑												Cellular Processes
Ribosome (ko03010)		↑											Genetic Information Processing
tRNA charging (TRNA-CHARGING-PWY)				↑								*L. crispatus, L. iners*	
DNA replication and recombination proteins						↑							
Transfer RNA biogenesis						↑							
Mitochondrial biogenesis						↑							
Cellular-associated protein families											↑		
Gene information processing pathways									↑				
General membrane transport pathways					↑								Membrane Transport
Phosphotransferase system (ko02060)		↑											
Coenzyme A biosynthesis III (PWY-4242)				↑								*L. crispatus, F. vaginae*	Metabolism of Cofactors and Vitamins
Coenzyme A biosynthesis II (COA-PWY-1)				↑								*L. crispatus, G. vaginalis and L . iners*	
Coenzyme A biosynthesis I (COA-PWY)				↑								*L. crispatus, L. iners*	
Folate transformation II (PWY-3841)				↑								*L. crispatus*	
S-adenosyl-L-methionine cycle I (PWY-6151)				↑								*L. crispatus, G. vaginalis*	
Pyrimidine DNA biosynthesis from CTP (PWY-7210).				↑								*L. rhamnosus and unclassified taxa*	Nucleotide Metabolism
Nucleotide biosynthesis through inosine-5'-phosphate biosynthesis (PWY-6123, PWY-6124, PWY-7234)								↑				*F. vaginae, G. vaginalis*	
5-aminoimidazole ribonucleotide biosynthesis (PWY-6277, PWY-6122)								↑				*F. vaginae, G. vaginalis*	
Pyrimidine biosynthesis pathways (PWY0-162, PWY-7211)								↑				*Unclassified taxa*	
Type II diabetes mellitus (ko04930)		↑											Human Diseases

Renal cell carcinoma (ko05211)							↑			↑			
Primary immunodeficiency (ko05340)		↑											
Signaling protein families					↑						↑		Signal Transduction
PPAR signaling pathway (ko03320)		↑											
Dioxin degradation (ko00621)	↑												Xenobiotics Biodegradation and Metabolism
Benzoate degradation (ko00362)	↑												
Xylene degradation (ko00622)	↑												
Styrene degradation (ko00643)	↑												
Aminobenzoate degradation (ko00627)		↑											
Acetylene degradation (P161-PWY)				↑								*L. jensenii, L.crispatus*	

*HPV—human papilloma virus; CC—cervical cancer; NC – normal controls. ↑ - significantly enriched pathway; ND – no difference.

Risk of bias assessment

Table S2. Risk of bias assessment (ROBINS-I modified according to the purpose of the study).

Study	Domain	Risk
**Hang Liu et al., 2022**	Confounding	**Moderate.** Study attempted to control for key confounders, including age, smoking, BMI, and menopausal status, however there might be other confounding factors that play a role (sexual activity, immune status, etc.).
	Participant selection	**Moderate.** Clear inclusion/exclusion criteria. Patients classified into groups after sequencing. No HPV negative control group.
	Exposure classification	**Low.** Exposure to HPV or cervical lesions was objectively measured using reliable methods like HPV genotyping and metagenomics.
	Departure from intended exposure	**Not applicable.** As a cross-sectional study with no interventions, this domain does not apply.
	Missing data	**Low.** The study does not mention any missing data issues.
	Outcome measurement	**Low.** Outcomes measured objectively. However, the study mentions that due to the contamination of human host DNA, effective sequencing data of vaginal microorganisms are limited, and more depth-sequencing methods that remove the human host genome are required to validate these results.
	Selection of reported result	**Low risk/unclear**. Registered study protocol not provided. Therefore, it is not clear whether selective reporting occurred. However, the study aims to comprehensively assess microbial diversity and no indication of selective reporting is apparent from the information provided.
	Overall risk of bias	**Moderate**

**Zheng et al., 2023**	Confounding	**Moderate.** Study seems to control for relevant confounders such as age, smoking, menopause, HPV infection, and other factors. However, additional details on the choice and adjustment fo r confounders in analysis are not provided clearly enough to fully assess the appropriateness of this control. Patients did not use antibiotics within 2 weeks and did not use vaginal medica tion or vaginal irrigation within 3 days and were matched by age and place of residence in dif ferent stage groups.
	Participant selecti on	**Low.** The study uses clearly defined groups and inclusion/exclusion criteria.
	Exposure classific ation	**Low.** The classification of different stages of cervical lesions and microbiota appears well-doc umented. Methods like metagenomic shotgun sequencing and TLR9 protein detection using W estern blotting were clearly described, suggesting accurate classification of exposures.
	Departure from i ntended exposur e	**Not applicable.** As a cross-sectional study with no interventions, this domain does not apply.
	Missing data	**Low.** The study does not mention any missing data issues.
	Outcome measurement	**Low.** Metagenomic sequencing is an objective outcome measurement tool.
	Selection of reported result	**Low risk/unclear**. Registered study protocol not provided. Therefore, it is not clear whether s elective reporting occurred. However, the study aims to comprehensively assess microbial div ersity and no indication of selective reporting is apparent from the information provided.
	Overall risk of bias	**Moderate**
**Hu et al., 20 24**	Confounding	**Moderate.** There might be other confounding factors that play a role (sexual activity, immune status, etc.). Genotype of hrHPV not equivalent in groups. Vaginal microbial samples may be regionally and ethnically heterogeneous, and only Chinese subjects were included in our stud y.
	Participant selection	**Low.** The study uses clearly defined groups, however, the selection is from available data of o ne other study.
	Exposure classification	**Low.** The study classifies participants into different disease stages (HPV-infected, CIN, CC) b ased on clinical data.
	Departure from intended exposure	**Not applicable.** As a cross-sectional study with no interventions, this domain does not apply.
	Missing data	**Low.** The study does not mention any missing data issues.
	Outcome measurement	**Low.** Metagenomic sequencing is an objective outcome measurement tool.
	Selection of reported result	**Low risk/unclear**. Registered study protocol not provided. Therefore, it is not clear whether s elective reporting occurred. However, the study aims to comprehensively assess microbial div ersity and no indication of selective reporting is apparent from the information provided.
	Overall risk of bias	**Moderate**
**Kwon et al., 2024**	Confounding	**Moderate.** Not all confounding factors accounted for.
	Participant selection	**Low.** Clear selection criteria, although the selected group is from another study. Small popula tion.
	Exposure classification	**Low.** The methods for diagnosing and classifying the stages of cervical disease are objective (h istological confirmation), which reduces the risk of misclassification bias.
	Departure from intended exposure	**Not applicable.**
	Missing data	**Low.** The study does not mention any missing data issues.
	Outcome measurement	**Low.** The study outcomes (microbiome composition and functional differences) are measured using standardized and widely accepted metagenomic sequencing techniques.

	Selection of reported result	**Low risk/unclear.** Registered study protocol not provided. Therefore, it is not clear whether s elective reporting occurred. However, the study aims to comprehensively assess microbial div ersity and no indication of selective reporting is apparent from the information provided.
	Overall risk of bias	**Moderate**
**Norenhag et al., 2024**	Confounding	**Moderate.** Important potential confounders like smoking, sexual activity, immune status, and other lifestyle factors that may influence both microbiota composition and cervical dysplasia a re not fully adjusted for.
	Participant selection	**Low.** Clear selection criteria. Participants were recruited from clinics and a cervical screening program. The matching process was used to balance characteristics like age, which minimizes selection bias.
	Exposure classification	**Low.** The classification of exposure (dysplasia or no dysplasia) was based on histopathologica l verification, which is a gold standard method for diagnosing cervical dysplasia.
	Departure from intended exposure	**Not applicable.** As a cross-sectional study with no interventions, this domain does not apply.
	Missing data	**Low.** The study does not mention any missing data issues.
	Outcome measurement	**Low.** The study outcomes (microbiome composition and functional pathways) were measure d using standardized, robust shotgun metagenomic sequencing methods.
	Selection of reported result	**Low risk/unclear**. Registered study protocol not provided. Therefore, it is not clear whether s elective reporting occurred. However, the study aims to comprehensively assess microbial div ersity and no indication of selective reporting is apparent from the information provided.
	Overall risk of bias	**Moderate**
**Fang et al., 2022**	Confounding	**Moderate.** There might be confounding factors that are not adjusted for. Women with recent a ntibiotic use, sexual activity, or other confounding conditions were excluded to minimize bias.
	Participant selection	**Low.** The study includes two clear groups: HR-HPV infected and uninfected women, selected based on consistent inclusion criteria.
	Exposure classification	**Low.** The classification of participants into HR-HPV positive and negative groups was done u sing reliable diagnostic tests, including HPV DNA testing and cytologic examination, which a re gold standards for detecting HR-HPV.
	Departure from intended exposure	**Not applicable.** As a cross-sectional study with no interventions, this domain does not apply.
	Missing data	**Low.** The study does not mention any missing data issues.
	Outcome measurement	**Low.** The study uses standardized and well-established techniques (16S rRNA gene sequencin g and metagenomic sequencing) to measure microbiota composition and functional pathways.
	Selection of reported result	**Low risk/unclear**. Registered study protocol not provided. Therefore, it is not clear whether s elective reporting occurred. However, the study aims to comprehensively assess microbial div ersity and no indication of selective reporting is apparent from the information provided.
	Overall risk of bias	**Moderate**
**Yang et al., 2020**	Confounding	**Moderate.** There might be confounding factors that are not adjusted for.
	Participant selection	**Low.** The study includes HPV16-positive women and HPV-negative controls from a colposco py clinic, with clear inclusion and exclusion criteria. Participants were recruited systematicall y from a specific population, minimizing the risk of selection bias.
	Exposure classification	**Low.** The classification of participants as HPV16-positive or HPV-negative was based on relia ble diagnostic methods, including HPV DNA testing and cytology.

Departure from intended exposure	**Not applicable.** As a cross-sectional study with no interventions, this domain does not apply.
Missing data	**Low.** The study does not mention any missing data issues.
Outcome measurement	**Low.** The study uses well-established shotgun metagenomic sequencing to measure the micro biota composition, providing high taxonomic and functional resolution.
Selection of reported result	**Low risk/unclear**. Registered study protocol not provided. Therefore, it is not clear whether s elective reporting occurred. However, the study aims to comprehensively assess microbial div ersity and no indication of selective reporting is apparent from the information provided.
Overall risk of bias	**Moderate**

Table S3. Search strategy

**Pubmed**<br>("vaginal microbiota"[MeSH] OR "vaginal flora" OR "vaginal microbial community" OR "genital tract microbiota" OR "vaginal dysbiosis") AND ("Cervical Dysplasia"[MeSH] OR "cervical intraepithelial neoplasia" OR "CIN" OR "cervical neoplasia" OR "precancerous cervical lesions") AND ("Human Papillomavirus"[MeSH] OR "HPV" OR "high-risk HPV" OR "persistent HPV" OR "oncogenic HPV") AND ("Metagenomics"[MeSH] OR "shotgun metagenomic sequencing" OR "metagenomic analysis" OR "next-generation sequencing" OR "NGS" OR "high-throughput sequencing")
**Web of science**<br>TS=("vaginal microbiota" OR "vaginal flora") AND TS=("cervical dysplasia" OR "cervical intraepithelial neoplasia" OR "CIN") AND TS=("HPV" OR "Human Papillomavirus") AND TS=("shotgun metagenomic sequencing" OR "metagenomics" OR "metagenomic analysis" OR "next-generation sequencing")
**SCOPUS**<br>TITLE-ABS-KEY(("vaginal microbiota" OR "vaginal flora" OR "vaginal microbial community" OR "genital tract microbiota" OR "vaginal dysbiosis")) AND TITLE-ABS-KEY(("cervical dysplasia" OR "cervical intraepithelial neoplasia" OR "CIN" OR "cervical neoplasia" OR "precancerous cervical lesions")) AND TITLE-ABS-KEY(("HPV" OR "Human Papillomavirus" OR "high-risk HPV" OR "persistent HPV" OR "oncogenic HPV")) AND TITLE-ABS-KEY(("shotgun metagenomic sequencing" OR "metagenomics" OR "metagenomic analysis" OR "next-generation sequencing" OR "NGS" OR "high-throughput sequencing"))
**ScienceDirect**<br>("vaginal microbiota" OR "vaginal flora" OR "vaginal microbial community" OR "genital tract microbiota" OR "vaginal dysbiosis") AND ("cervical dysplasia" OR "cervical intraepithelial neoplasia" OR "CIN" OR "cervical neoplasia" OR "precancerous cervical lesions") AND ("HPV" OR "Human Papillomavirus" OR "high-risk HPV" OR "persistent HPV" OR "oncogenic HPV") AND ("shotgun metagenomic sequencing" OR "metagenomics" OR "metagenomic analysis" OR "next-generation sequencing" OR "NGS" OR "high-throughput sequencing")
